# INFOGEST Digestion and In Vitro Rat Fecal Fermentation of *Bletilla striata* Polysaccharides: Digestive Stability and Dose-Specific Microbiota–Metabolite Responses

**DOI:** 10.3390/foods15213803

**Published:** 2026-10-23

**Authors:** Junliang Liu, Jiayao Lu, Peiyi Wang, Chenglin Zhu, Luca Laghi

**Affiliations:** 1 College of Pharmacy and Food, Southwest Minzu University, Chengdu 610041, China; 2 Key Laboratory of Research and Application of Ethnic Medicine Processing and Preparation on the Qinghai Tibet Plateau, Southwest Minzu University, Chengdu 610041, China; 3 Sichuan Zoige Alpine Wetland Ecosystem National Observation and Research Station, Southwest Minzu University, Chengdu 610041, China; 4 Department of Agricultural and Food Sciences, University of Bologna, 47521 Cesena, Italy; l.laghi@unibo.it

**Keywords:** *Bletilla striata* polysaccharides, dose-specific response, in vitro, gut microbiota, metabolomics

## Abstract

Natural polysaccharides have considerable potential to modulate the gut microbial ecosystem. This study integrated INFOGEST-based simulated gastrointestinal digestion, in vitro fermentation using rat feces, untargeted metabolomics, and 16S rRNA sequencing to characterize the digestive stability of *Bletilla striata* polysaccharides (BSP) and the dose-specific microbial and metabolic responses to BSP. During digestion, BSP exhibited changes in total and reducing sugar content while retaining appreciable antioxidant activity. During fermentation, BSP promoted microbial growth and reduced pH. The medium dose selectively enriched *Bifidobacterium*, whereas the high dose favored *Lactobacillus* and *Ligilactobacillus*, accompanied by lower relative abundances of potentially unfavorable taxa, including *Escherichia–Shigella* and *Proteus*. Metabolomic analysis further revealed that BSP predominantly altered amino acid, aromatic amino acid, and carbohydrate metabolism, generating distinct dose-specific metabolic signatures. Overall, BSP remained available for microbial utilization after digestion and reshaped both microbial composition and metabolic profiles in a dose-specific manner, indicating its promise as a functional food ingredient.

## 1. Introduction

*Bletilla striata*, an orchid used in traditional Chinese medicine, has historically been employed to control bleeding and swelling and to promote tissue repair. Pharmacological research has subsequently documented anti-inflammatory, antioxidant, antimicrobial, immunoregulatory, and wound-repair activities [1]. Much of this interest has focused on *Bletilla striata* polysaccharides (BSP), which have been associated with antioxidant, anti-inflammatory, immunomodulatory, hemostatic, and gastrointestinal protective effects and have possible uses in food, pharmaceutical, and biomaterial formulations [2]. We isolated a water-soluble fraction from *B. striata* tubers that contained 63.46% carbohydrate and had number- and weight-average molecular weights (Mn and Mw) of 6.75 × 10^4^ and 3.73 × 10^5^ g/mol, respectively [3]. Those results motivated the present assessment of its behavior during digestion and its availability to intestinal microorganisms.

Following oral ingestion, polysaccharides are exposed to sequential gastrointestinal environments that may alter their stability and physicochemical properties. The standardized static in vitro digestion model described by the INFOGEST initiative has been extensively used to characterize the digestive behavior of food components in the upper gastrointestinal tract [4,5]. Because many structurally complex polysaccharides are only partially degraded by host digestive enzymes, substantial fractions may reach the colon and become substrates for microbial fermentation [6]. Recent studies further indicate that the gastrointestinal stability of medicinal and edible polysaccharides is highly source-dependent. *Polygonatum cyrtonema* polysaccharide underwent partial degradation during simulated gastrointestinal digestion, accompanied by an increase in reducing sugar content and a slight reduction in molecular weight [7]. In contrast, polysaccharides from nine *Polygonatum* species remained largely resistant to salivary, gastric, and intestinal digestion but were subsequently degraded and utilized during fecal fermentation [8]. Similarly, polysaccharides derived from *Cordyceps sinensis* and *Lycium barbarum* showed high resistance to upper gastrointestinal digestion but were rapidly degraded during subsequent human fecal fermentation [9]. The digestive stability of these polysaccharides may reflect glycosidic linkages that are poorly hydrolyzed by host digestive enzymes. For example, α-amylase acts primarily on α-(1→4) glucosidic bonds, whereas other linkage types may resist its action. Acid-sensitive linkages may nevertheless undergo partial cleavage in the stomach [10]. These differences in susceptibility to enzymatic and acid hydrolysis may help explain the contrasting digestion profiles and influence the carbohydrates available for colonic fermentation.

Emerging evidence has begun to clarify the gastrointestinal digestion and colonic fermentation characteristics of BSP. Wang et al. reported that BSP underwent partial degradation during simulated salivary digestion, whereas its molecular weight remained relatively stable during both the gastric and the small-intestinal phases. Following in vitro fecal fermentation, BSP was used by the microorganisms of the gut, accompanied by changes in the composition of the microbial community and by an increase in the production of short-chain fatty acids [11]. However, this study primarily compared BSP with its corresponding oligosaccharides and did not address dose as an ecological variable shaping microbial selection and metabolic output. This limitation is particularly relevant because fermentable carbohydrate–microbiota interactions are not necessarily proportional to substrate concentration. Different inulin supplementation levels have been shown to alter both the rate of microbial succession and the composition of responsive taxa [12], while varying the concentration of pectin-based dietary fibers can modify substrate degradation kinetics, microbial biomass, fermentation metabolites, and lactate cross-feeding [13]. Dose-dependent differences in microbial communities and metabolic outputs have likewise been reported for type 2 resistant starch [14]. Metabolomic studies of jujube polysaccharides and ulvan have revealed fermentation-associated metabolic changes [15,16]. Structurally distinct pectins also elicit different microbial and metabolic responses [17], highlighting the value of linking taxonomic shifts to metabolic outputs. Despite these advances, whether different BSP doses establish distinct bacterial communities and metabolomic profiles and how these dose-specific bacterial shifts are associated with key metabolites remain unresolved.

To clarify these unresolved questions, this work systematically studied the gastrointestinal digestion and dose-specific microbial fermentation of BSP using an integrated in vitro approach. Firstly, BSP underwent simulated oral, gastric, and intestinal digestion to quantify the modifications in total and reducing sugars and in antioxidant activity. Subsequently, an in vitro model for fecal fermentation was used to compare the effects of low, medium, and high BSP doses on microbial growth, fermentation pH, and gut microbial community composition. 16S rRNA sequencing and untargeted metabolomics were further integrated to characterize dose-specific microbial and metabolic responses, followed by microbiota–metabolite correlation analysis to identify potential associations between key bacterial taxa and representative metabolites. Collectively, this study aimed to clarify how BSP is transformed during gastrointestinal digestion and subsequently utilized by the gut microbiota, with particular emphasis on whether different BSP doses generate distinct microbial and metabolic phenotypes. These findings provide integrated microbiological and metabolic evidence for understanding the potential prebiotic properties of BSP and support its rational development as a functional food ingredient.

## 2. Materials and Methods

### 2.1. Materials and Reagents

*Bletilla striata* samples were purchased from a traditional Chinese medicinal herb market in Kunming, Yunnan Province, China. α-Amylase, pepsin, and pancreatic lipase were supplied by Shanghai Yuanye Biotechnology Co., Ltd. (Shanghai, China). Porcine bile salts, tryptone, peptone, and yeast extract were sourced from Beijing Solepol Technology Co., Ltd. (Beijing, China). Glucose was supplied by Chengdu Jinshan Chemical Reagent Co., Ltd. (Chengdu, China). 3,5-dinitrosalicylic acid (DNS) reagent was sourced from Guangzhou Howei Pharma Tech Co., Ltd. (Guangzhou, China). 2,2-diphenyl-1-picrylhydrazyl (DPPH) was supplied by Phygene Life Sciences Co., Ltd. (Fuzhou, China). ABTS diammonium salt (2,2′-azino-bis(3-ethylbenzothiazoline-6-sulfonic acid) diammonium salt), hemin, L-cysteine, vitamin K, resazurin, and L-cysteine hydrochloride were supplied by Shanghai Aladdin Biochemical Technology Co., Ltd. (Shanghai, China). Methanol and acetonitrile were sourced from ANPEL Laboratory Technologies Inc. (Shanghai, China). Ammonium acetate was supplied by Sigma-Aldrich (Shanghai) Trading Co., Ltd. (Shanghai, China), and ammonia solution was sourced from Thermo Fisher Scientific (Waltham, MA, USA). The remaining reagents were analytical-grade products.

### 2.2. Preparation of BSP

BSP was produced following the procedure reported by Zhang et al. [3]. In detail, *Bletilla striata* tubers were dried at 45 °C until their mass stabilized, milled, and sieved through a 30-mesh screen. The powder was soaked in 95% ethanol for 30 min at a solid-to-liquid ratio of 1:5 (*w*/*v*), followed by extraction with 95% ethanol (5 mL/g) and petroleum ether (5 mL/g; boiling range, 60–90 °C) at 70 °C for 2 h. After filtration and drying, the resulting powder was suspended in water at a solid-to-liquid ratio of 1:40 (*w*/*v*) and extracted at 70 °C for 2 h under continuous stirring at 150 rpm. Two aqueous extraction cycles were completed, after which both extracts were pooled.

The pooled aqueous extract was concentrated, treated with Sevag reagent to remove protein, and separated to recover the aqueous layer. The deproteinized extract was further concentrated, and anhydrous ethanol was added to achieve a final ethanol concentration of 80% (*v*/*v*). Polysaccharides were allowed to precipitate for 12 h at 4 °C. The resulting precipitate was sequentially washed with anhydrous ethanol, acetone, and ether and then dried at 30 °C to constant weight to obtain crude BSP. The crude polysaccharides were subsequently purified using a DEAE-52 cellulose column to obtain purified BSP. The purified BSP contained 63.46% total carbohydrates, with a number-average molecular weight (Mn) of 6.75 × 10^4^ g/mol and a weight-average molecular weight (Mw) of 3.73 × 10^5^ g/mol.

### 2.3. In Vitro Simulated Salivary and Gastrointestinal Digestion

Digestion was simulated with the static INFOGEST protocol [4], with minor adaptations. Simulated salivary fluid (SSF), simulated gastric fluid (SGF), and simulated intestinal fluid (SIF) were made beforehand as 1.25-fold stock solutions, with 400 mL prepared for each fluid.

For oral digestion, BSP solution (10 mg/mL) was mixed with SSF, salivary amylase (3700 U/mL), CaCl_2_·2H_2_O, and ultrapure water to achieve a final BSP-to-digestion mixture ratio of 1:1 (*v*/*v*). The final concentrations of CaCl_2_ and salivary amylase were adjusted to 1.5 mM and 75 U/mL, respectively. This oral-phase mixture remained at 37 °C for 2 min. Samples were collected at 0, 1, and 2 min and heated at boiling temperature for 5 min to stop enzyme activity. Once cooled to room temperature, samples were centrifuged for 10 min at 3500 rpm; the recovered supernatants were kept at −20 °C pending analysis.

For gastric digestion, the oral digesta was mixed with SGF, pepsin (2000 U/mg), CaCl_2_·2H_2_O, and ultrapure water at a final oral-digesta-to-gastric-digestion mixture ratio of 1:1 (*v*/*v*), with SGF added at 0.8 times the volume of the oral digesta. The pH was immediately adjusted to 3.0 with 1 M HCl. The final concentrations of CaCl_2_ and pepsin were 0.15 mM and 2000 U/mL, respectively. Incubation then proceeded for 2 h at 37 °C. Samples were collected at 1 and 2 h and heated at boiling temperature for 5 min to stop enzyme activity. Once cooled to room temperature, samples were centrifuged for 10 min at 3500 rpm; the recovered supernatants were kept at −20 °C pending analysis.

For intestinal digestion, the gastric digesta was mixed with SIF, bile salts, pancreatic enzymes (100 U/mg), CaCl_2_·2H_2_O, and ultrapure water at a final gastric-digesta-to-intestinal-digestion mixture ratio of 1:1 (*v*/*v*), with SIF added at 0.8 times the volume of the gastric digesta. The pH was adjusted to 7.0 using 5 M NaOH. The final concentrations of bile salts, CaCl_2_, and pancreatic enzymes were 10 mM, 0.6 mM, and 100 U/mL, respectively. Incubation then proceeded for 2 h at 37 °C. Samples were collected at 1 and 2 h and heated at boiling temperature for 5 min to stop enzyme activity. Once cooled to room temperature, samples were centrifuged for 10 min at 3500 rpm; the recovered supernatants were kept at −20 °C pending analysis.

### 2.4. Determination of Reducing Sugar and Total Sugar Content

After simulated digestion, reducing sugars were quantified by the DNS assay according to Hu et al. [18]. Digesta samples (1 mL) collected at different time points during simulated oral, gastric, and intestinal digestion were mixed with 2 mL of DNS reagent and heated in a boiling water bath for 5 min. After cooling, the mixture was diluted to 15 mL with distilled water and thoroughly mixed. Absorbance readings at 540 nm were converted to reducing-sugar concentrations with the relevant calibration curve.

The total sugar content of the digesta was determined using the phenol–sulfuric acid method after minor adaptation [19]. In detail, 0.6 mL of digesta collected at different time points during simulated oral, gastric, and intestinal digestion was mixed with 0.4 mL of 6% phenol solution in an ice-water bath. After mixing, 1 mL of concentrated sulfuric acid was rapidly added, and the mixture was immediately vortexed and incubated at room temperature for 30 min. A microplate reader provided the 490-nm absorbance, from which total sugar was obtained using the appropriate calibration curve.

The calibration equations were Y = 0.00143x − 0.00019 (*R*^2^ = 0.9963) for total sugar and Y = 1.3942x + 0.0001 (*R*^2^ = 0.9971) for reducing sugar.

### 2.5. Determination of Antioxidant Activity of Digesta

The antioxidant activity of the digesta was assessed with DPPH, ABTS^+^, and hydroxyl-radical scavenging assays following the procedure of Ma et al. [20], with minor adaptations.

For the DPPH radical scavenging assay, 1 mL of digesta was mixed with 2 mL of DPPH solution (0.20 mM). Following 30 min at 30 °C without light exposure, absorbance was read at 517 nm. Distilled water was used instead of the sample for the blank (*A*_0_), whereas anhydrous ethanol was used instead of the DPPH solution for the control (*A*_2_). The DPPH radical scavenging activity was calculated using Equation (1):

(1) $$\text{DPPH radical scavenging rate}\;\left(\%\right)=\left(1-\frac{A_{1}-A_{2}}{A_{0}}\right)\times 100\%$$

where *A*_0_, *A*_1_, and *A*_2_ denote, respectively, the absorbance of the blank, sample, and control.

For the ABTS^+^ radical scavenging assay, identical volumes of ABTS solution (7.40 mM) and potassium persulfate solution (2.60 mM) were combined and left for 16 h at room temperature in darkness to form the ABTS^+^ stock solution. The stock solution was then diluted with anhydrous ethanol to an absorbance of 0.70 ± 0.02 at 734 nm. Subsequently, 1 mL of digesta was mixed with 2 mL of the diluted ABTS^+^ solution and incubated at room temperature in the dark for 6 min. Absorbance was recorded at 734 nm. Distilled water was used instead of the sample for the blank (*A*_0_). The ABTS^+^ radical scavenging activity was calculated using Equation (2):

(2) $${\mathrm{ABTS}}^{+}\;\text{radical scavenging rate}\;\left(\%\right)=\frac{A_{0}-A_{1}}{A_{0}}\times 100\%$$

where *A*_0_ and *A*_1_ represent the absorbance values of the blank and sample, respectively.

For the hydroxyl radical scavenging assay, 1 mL of digesta was mixed with 2.0 mL of salicylic acid in ethanol (9.0 mM), 2.0 mL of FeSO_4_ solution (9.0 mM), and 0.5 mL of H_2_O_2_ solution (10.0 mM). The mixture was incubated at 37 °C for 30 min, and the absorbance was measured at 510 nm. Distilled water was used instead of the sample for the blank (A_0_), whereas distilled water was used instead of H_2_O_2_ solution for the control (*A*_2_). The hydroxyl radical scavenging activity was calculated using Equation (3):

(3) $$\text{Hydroxyl radical scavenging rate}\;\left(\%\right)=\left(1-\frac{A_{1}-A_{2}}{A_{0}}\right)\times 100\%$$

where *A*_0_, *A*_1_, and *A*_2_ represent the absorbance values of the blank, sample, and control, respectively.

### 2.6. Fecal Collection

The fecal sample collection protocol was approved by the Institutional Animal Care and Use Committee (IACUC) of Southwest Minzu University (approval no. SMU-202401148).

Rat fecal inocula were selected to allow control of donor background when comparing fermentation responses to different BSP doses, consistent with previous studies [21,22,23,24]. All donor rats were of the same strain and maintained on the same diet under the same housing conditions. Sterile forceps and centrifuge tubes were used to obtain fresh feces from 10 healthy rats, following the procedure of Teng et al. [25], with slight modifications. Equivalent fecal masses from the 10 rats were combined aseptically into a single pool and mixed with sterile modified physiological saline containing 0.5 g/L L-cysteine hydrochloride and 9.0 g/L NaCl to prepare a 20% (*w*/*v*) fecal suspension. After centrifugation at 500× *g* and 4 °C for 5 min, the supernatant served as inoculum for fermentation.

### 2.7. Preparation of the Basal Medium

The basal medium was prepared following the procedure of Liu et al. [26] and had the following composition: 3 g/L tryptone, 2 g/L peptone, 2 g/L yeast extract, 0.1 g/L NaCl, 0.04 g/L K_2_HPO_4_, 0.04 g/L KH_2_PO_4_, 0.01 g/L MgSO_4_·7H_2_O, 0.01 g/L CaCl_2_·2H_2_O, 2 g/L NaHCO_3_, 0.02 g/L hemin, 0.5 g/L cysteine-HCl, 0.5 g/L bile salts, 2.0 mL/L Tween 80, 10 μL/L vitamin K, and 1 mL/L of 1% resazurin solution. Before use, the medium was brought to pH 7.0 and sterilized for 20 min at 121 °C.

### 2.8. In Vitro Anaerobic Fermentation

Through adaptations of the method of Zhou et al. [27], BSP was sterilized by UV irradiation before fermentation and placed in a sterile plate until use. Different amounts of BSP were added to the sterilized basal medium to establish three dose groups: low-dose BSP (LB, 2 mg/mL), medium-dose BSP (MB, 6 mg/mL), and high-dose BSP (HB, 10 mg/mL). A blank control containing only rat fecal inoculum and basal medium was included. The fermentation medium of each group was mixed with the pooled fecal inoculum at a volume ratio of 9:1 and dispensed into five separate anaerobic fermentation tubes. The same pooled inoculum was used for all four groups. Fermentation was conducted at 37 °C under anaerobic conditions in the dark for 48 h. Samples were collected at 0, 6, 12, 24, and 48 h and immediately stored at −80 °C until further analysis.

### 2.9. Measurement of Bacterial Amount and pH

Bacterial biomass and pH were monitored in samples collected from the same five fermentation tubes per group at 0, 6, 12, 24, and 48 h. Biomass was estimated as optical density at 600 nm (OD_600_), and pH was read with a pH meter.

### 2.10. Microbial Diversity Analysis

At 48 h, community DNA was isolated separately from each of the five fermentation tubes per group using the E.Z.N.A.^®^ soil DNA Kit (Omega Bio-Tek, Norcross, GA, USA), and DNA quality was assessed. The bacterial 16S rRNA V3-V4 region was PCR-amplified with primers 338F (5′-ACTCCTACGGGAGGCAGCAG-3′) and 806R (5′-GGACTACHVGGGTWTCTAAT-3′). Amplicons were resolved on 2% agarose, excised and cleaned with a gel-extraction kit, and quantified on a Qubit 4.0. Sequencing libraries were constructed using the NEXTFLEX Rapid DNA-Seq Kit (Bioo Scientific Corporation, Austin, TX, USA) and sequenced on the Illumina PE300 platform (Illumina, Inc., San Diego, CA, USA). Fastp was used for quality control of paired-end reads, which were subsequently joined with FLASH. Based on QIIME 2, the merged optimized sequences were denoised using the DADA2 plugin to obtain amplicon sequence variants (ASVs). Reads assigned to chloroplasts or mitochondria were excluded. Sequencing depth was standardized by rarefying each sample to 20,000 reads. After rarefaction, the average Good’s coverage of all samples reached 1. Taxonomic classification of ASVs was performed using the Naive Bayes classifier in QIIME 2 against the SILVA 16S rRNA gene database (v138).

### 2.11. Untargeted Metabolomics Analysis

Samples collected at 48 h from the same five fermentation tubes per group used for metabolomic analysis were placed separately in clean microcentrifuge tubes, and an extraction solvent containing isotope-labeled internal standards was added. Samples were vortexed for 30 s and then sonicated to thoroughly disrupt the samples and release metabolites. Subsequently, the samples were kept at −40 °C for 1 h. After standing, the samples were centrifuged at high speed for 15 min. The resulting supernatant was placed in autosampler vials for LC-MS. In addition, to evaluate method stability and reliability, equal volumes of each sample supernatant were mixed to prepare quality control (QC) samples, which were analyzed together with the test samples. Metabolites were separated on a Vanquish ultra-high-performance liquid chromatography (UHPLC) system (Thermo Fisher Scientific, Waltham, MA, USA) equipped with a Waters ACQUITY UPLC BEH Amide column (2.1 mm × 50 mm, 1.7 μm, Waters Corporation, Milford, MA, USA). Mobile phase A was water containing 25 mmol/L ammonium acetate and 25 mmol/L ammonium hydroxide, and mobile phase B was acetonitrile. The column temperature was 30 °C, and the flow rate was 0.50 mL/min. The gradient was programmed at 95% B from 0.00 to 0.25 min, 65% B at 3.50 min, and 40% B at 4.00 min. It remained at 40% B until 4.50 min, returned to 95% B at 4.55 min, and was maintained at 95% B until 6.00 min. The autosampler temperature was set at 4 °C, and the injection volume was 2 μL. Full-scan MS data were acquired over m/z 70–1200 using an Orbitrap Exploris 120 mass spectrometer (Thermo Fisher Scientific, Bremen, Germany) controlled by Xcalibur software (version 4.4). Full-scan MS and MS/MS resolutions were 60,000 and 15,000, respectively, with stepped normalized collision energies of 20, 30, and 40. The sheath and auxiliary gas flow rates were 50 and 15 arbitrary units, respectively, and the capillary temperature was 320 °C. Spray voltages were 3.8 kV in positive ion mode and −3.4 kV in negative ion mode.

Raw data were converted to mzXML format using ProteoWizard (version 3.0.24054). Metabolite identification was performed using R-based software (version 4.4.3) and BiotreeDB (version 3.0). Feature-level filtering was based on relative standard deviation (RSD). For missing-value filtering, features with no more than 50% missing values within a group or across all groups were retained. Remaining missing values were imputed using half the minimum value, and peak areas were normalized to the total ion current (TIC) of each sample. Only metabolites assigned Level 1 identification confidence were included in the analyses reported in this study. Level 1 assignments required matching precursor-ion m/z (MS1), MS/MS spectra (MS2), and retention time to reference data from authentic standards in BiotreeDB.

### 2.12. Statistical Analysis

Results are presented as means ± standard deviations (SDs). One-way analysis of variance (ANOVA), followed by Tukey’s post hoc test, was conducted using SPSS version 26.0. Statistical significance was defined as *p* < 0.05 unless otherwise specified. Bar charts and doughnut charts were generated using Origin 2021.

Microbial community structure was explored using principal component analysis (PCA) and hierarchical clustering. Differences among groups were assessed using analysis of similarities (ANOSIM) based on Euclidean distances, with 999 permutations. Bacterial relative abundances were compared using Kruskal–Wallis tests, with false discovery rate (FDR) correction across all tested taxa. Taxa with FDR-adjusted *p* < 0.05 were considered significantly different among groups. Linear discriminant analysis effect size (LEfSe) was used to identify discriminative taxa with linear discriminant analysis (LDA) scores > 4.0. Microbial data were visualized using the Majorbio Cloud Platform (https://v.majorbio.com/).

Metabolomic profiles were explored using PCA and partial least squares discriminant analysis (PLS-DA). PLS-DA and Kyoto Encyclopedia of Genes and Genomes (KEGG) pathway enrichment analyses were performed using OmicStudio (www.omicstudio.cn). The PLS-DA model was evaluated by permutation testing. Each BSP dose group was compared with Blank using two-sided Welch’s *t*-tests on log_2_-transformed abundances. Benjamini–Hochberg correction was applied across all tested metabolites within each comparison. Differential metabolites were then selected using |log_2_FC| > 1 and FDR-adjusted *p* < 0.05. Volcano plots were generated using www.bioinformatics.com.cn. UpSet plots and clustering heatmaps of differential metabolites were generated using the Majorbio Cloud Platform.

Associations between differential bacterial genera and selected metabolites were examined using Spearman’s rank correlation. Correlation heatmaps were generated using ChiPlot (https://www.chiplot.online/).

## 3. Results

### 3.1. Changes in Total Sugar and Reducing Sugar Content During Simulated Digestion

The changes in total sugar and reducing sugar content of BSP during in vitro simulated digestion were determined (Table 1). During the simulated salivary phase (SSF), the total sugar content remained relatively stable, with no significant differences among sampling time points (*p* > 0.05). The reducing sugar content also showed no significant change, ranging from 0.481 ± 0.014 to 0.498 ± 0.005 mg/mL.

During simulated gastric digestion (SGF), the total sugar content decreased from 0.599 ± 0.009 mg/mL at 1 h to 0.591 ± 0.017 mg/mL at 2 h and was significantly lower than that during the salivary phase (*p* < 0.05). In contrast, the reducing sugar content increased slightly during gastric digestion, reaching 0.533 ± 0.009 mg/mL and 0.534 ± 0.014 mg/mL at 1 and 2 h, respectively, both significantly higher than the values observed during the salivary phase (*p* < 0.05).

During simulated intestinal digestion (SIF), the total sugar content decreased further to 0.533 ± 0.010 mg/mL at 1 h and 0.528 ± 0.004 mg/mL at 2 h, both significantly lower than those observed during the salivary and gastric phases (*p* < 0.05). Meanwhile, the reducing sugar content decreased to 0.428 ± 0.002 mg/mL and 0.428 ± 0.006 mg/mL at 1 and 2 h, respectively, and was significantly lower than that during the gastric phase (*p* < 0.05).

Overall, the total sugar content of BSP progressively decreased as digestion proceeded, whereas the reducing sugar content increased during gastric digestion and subsequently declined during the intestinal phase, indicating distinct stage-dependent changes during simulated gastrointestinal digestion.

### 3.2. Changes in the Antioxidant Properties of BSP During Simulated Gastrointestinal Digestion

**Figure 1:**
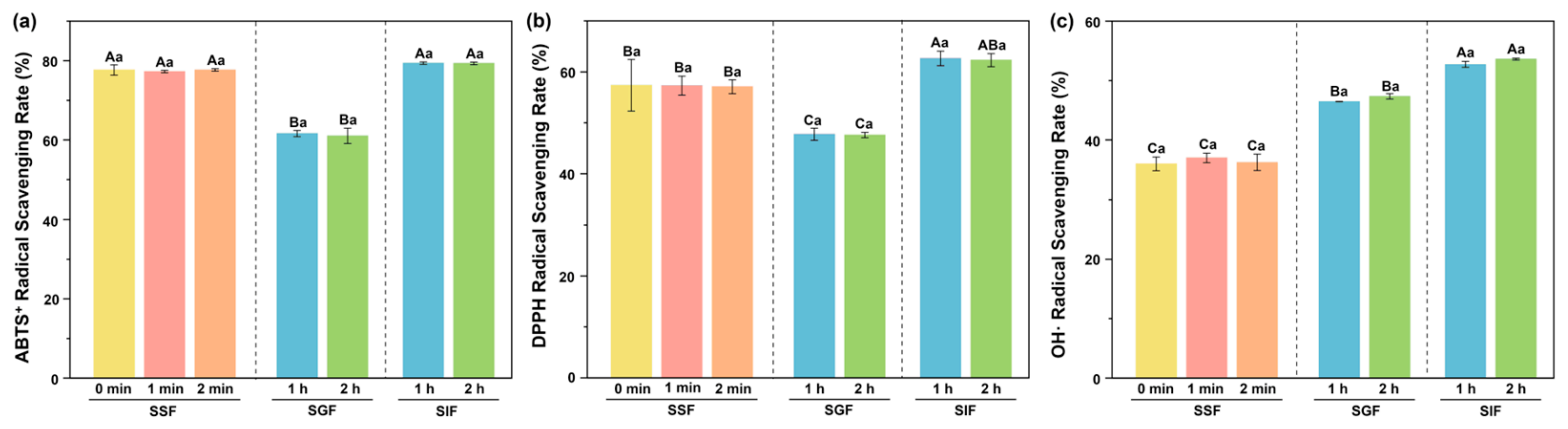
Changes in the antioxidant properties of BSP during simulated gastrointestinal digestion: (**a**) ABTS^+^ radical scavenging activity, (**b**) DPPH radical scavenging activity, and (**c**) •OH radical scavenging activity. Different uppercase letters indicate significant differences among digestion phases, whereas different lowercase letters indicate significant differences among sampling times within the same digestion phase (*p* < 0.05).

As shown in Figure 1, the antioxidant activity of BSP exhibited distinct changes during in vitro simulated digestion. During SSF, no significant changes were observed in the scavenging activities against ABTS^+^, DPPH, or •OH (*p* > 0.05), indicating that the antioxidant activity of BSP remained relatively stable during this stage. During SGF, the ABTS^+^ scavenging activity decreased from approximately 77% during SSF to approximately 62% at both 1 and 2 h (*p* < 0.05). Similarly, DPPH scavenging activity decreased from approximately 57% during SSF to approximately 48% during SGF (*p* < 0.05). In contrast, •OH scavenging activity increased from approximately 36% during SSF to approximately 48% at both 1 and 2 h of SGF (*p* < 0.05). During SIF, the ABTS^+^ scavenging activity increased significantly compared with that during SGF (*p* < 0.05). DPPH scavenging activity reached approximately 63% at both 1 and 2 h, significantly higher than that observed during SGF (*p* < 0.05). The •OH scavenging activity increased further to approximately 53% and 54% at 1 and 2 h, respectively, and was significantly higher than that during both SSF and SGF (*p* < 0.05). Overall, ABTS^+^ and DPPH scavenging activities decreased during SGF and subsequently recovered during SIF, whereas •OH scavenging activity increased progressively as digestion proceeded.

### 3.3. Effects of Different BSP Doses on OD_600_ and pH During In Vitro Fermentation

**Figure 2:**
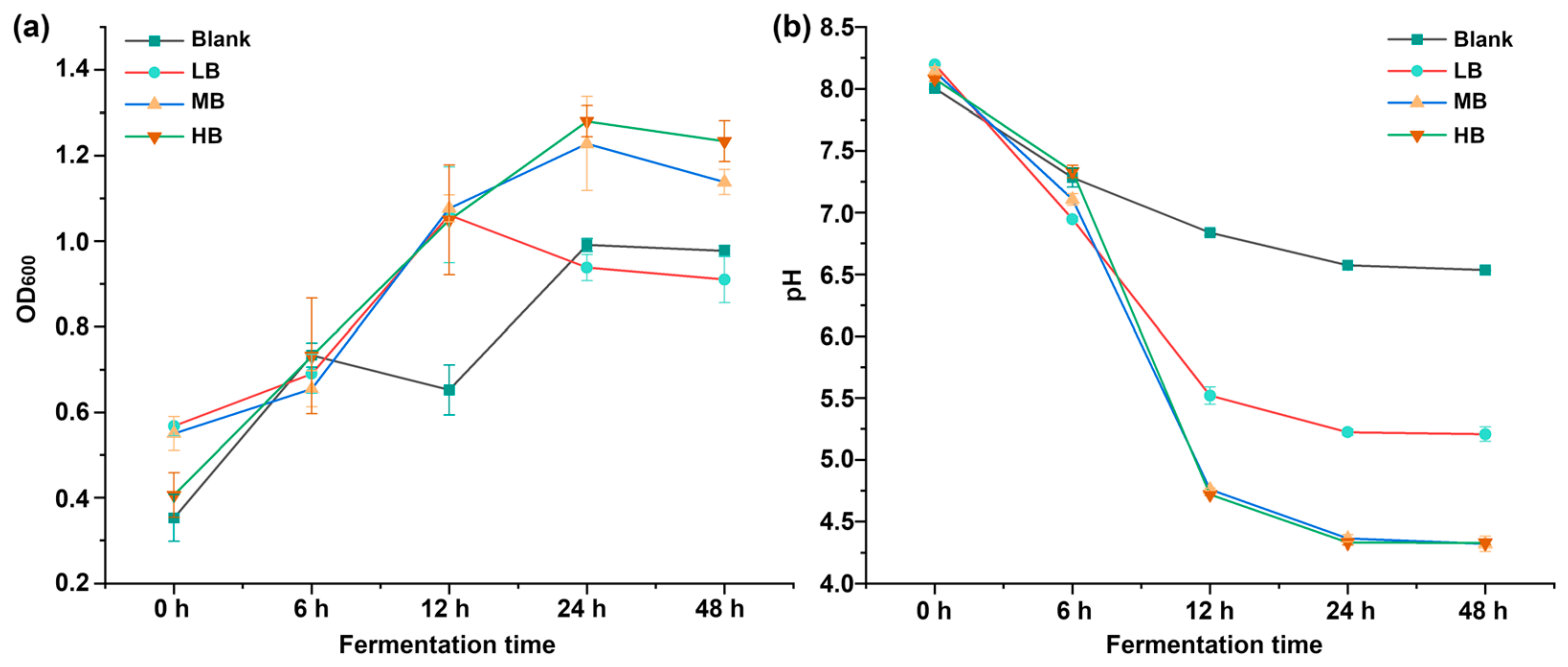
Effects of different BSP doses on OD_600_ and pH during in vitro fermentation: (**a**) changes in OD_600_; (**b**) changes in pH. *n* = 5 fermentation tubes per group; the same tubes were sampled at all time points.

As shown in Figure 2a, OD_600_ values in all groups initially increased and then tended to stabilize during fermentation. At 0 h, the OD_600_ values of the Blank, LB, MB, and HB groups were approximately 0.35, 0.56, 0.55, and 0.40, respectively, and increased markedly after 6 h. At 12 h, the OD_600_ values of the LB, MB, and HB groups reached approximately 1.05, 1.07, and 1.06, respectively, all higher than those of the Blank group (approximately 0.65). At 24 h, the OD_600_ values of the MB and HB groups increased to approximately 1.23 and 1.27, respectively, exceeding those of the LB (approximately 0.94) and Blank (approximately 0.99) groups. At 48 h, relatively high OD_600_ values were maintained in the MB and HB groups, at approximately 1.14 and 1.23, whereas those in the LB and Blank groups were approximately 0.91 and 0.98, respectively.

As shown in Figure 2b, the pH decreased progressively in all groups throughout fermentation. The initial pH values were approximately 8.0–8.2. From 12 h onward, the decrease in pH was more pronounced in the BSP-treated groups. At 24 and 48 h, the pH values of all BSP-treated groups decreased further and tended to stabilize, whereas the Blank group remained at relatively higher pH values of approximately 6.58 and 6.54, respectively. Among the BSP-treated groups, the MB and HB groups exhibited lower pH values at the later fermentation stages.

Overall, BSP supplementation altered both OD_600_ and pH during fermentation, with the MB and HB groups showing higher OD_600_ values and lower pH levels at the later stages of fermentation.

### 3.4. Effects of Different BSP Doses on Microbial Diversity During In Vitro Fermentation

*α-* and *β*-diversity analyses were performed to evaluate changes in microbial community diversity and structure following fermentation with different BSP doses. Alpha diversity was assessed using the Chao1, Shannon, Simpson, and Coverage indices.

**Figure 3:**
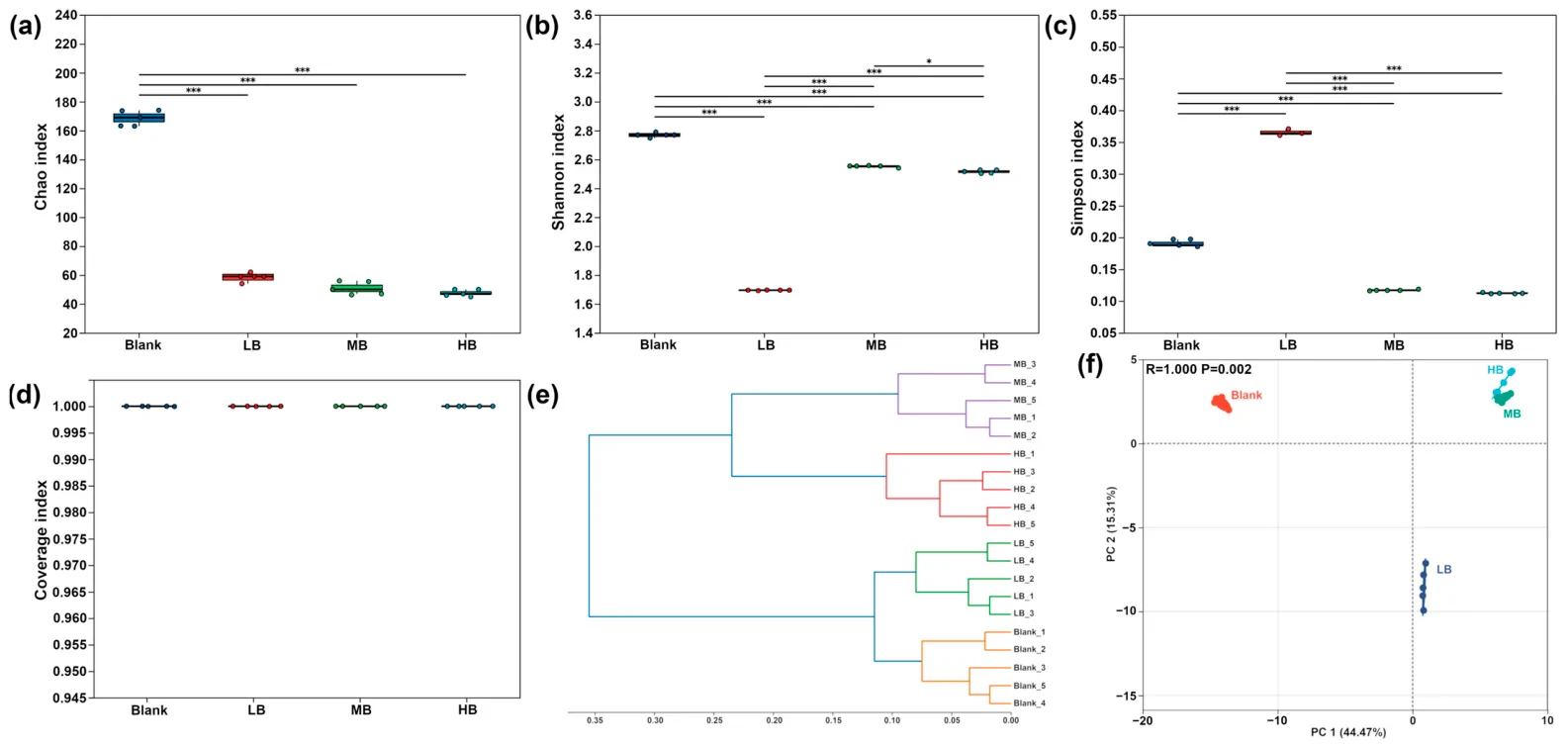
Effects of different BSP doses on microbial community diversity during in vitro fermentation: (**a**) Chao1 index, (**b**) Shannon index, (**c**) Simpson index, (**d**) Coverage index, (**e**) hierarchical clustering analysis, and (**f**) principal component analysis (PCA) of microbial community structure. Significant differences between groups are indicated by * *p* < 0.05 and *** *p* < 0.001. Analyses were based on samples from five separate fermentation tubes per group, all inoculated from the same pooled fecal sample.

As shown in Figure 3a–d, BSP supplementation markedly altered the α-diversity profiles of the microbial communities. Compared with the Blank group, the Chao1 index was significantly lower in the LB, MB, and HB groups. The Shannon index was lowest in the LB group but was higher in the MB and HB groups. The Simpson index also varied among the BSP-treated groups. Coverage values were close to 1.0 for all samples, indicating sufficient sequencing depth.

Hierarchical clustering and PCA showed distinct microbial community structures among the Blank, LB, MB, and HB groups (Figure 3e,f). ANOSIM further supported significant differences among groups (*R* = 1.000, *p* = 0.002).

Overall, different BSP doses produced distinct changes in microbial community diversity and structure during in vitro fermentation.

### 3.5. Effects of Different BSP Doses on Microbial Community Composition During In Vitro Fermentation

To further evaluate the effects of different BSP doses on fecal microbial composition, taxonomic profiles at the phylum and genus levels were analyzed based on 16S rRNA sequencing data.

**Figure 4:**
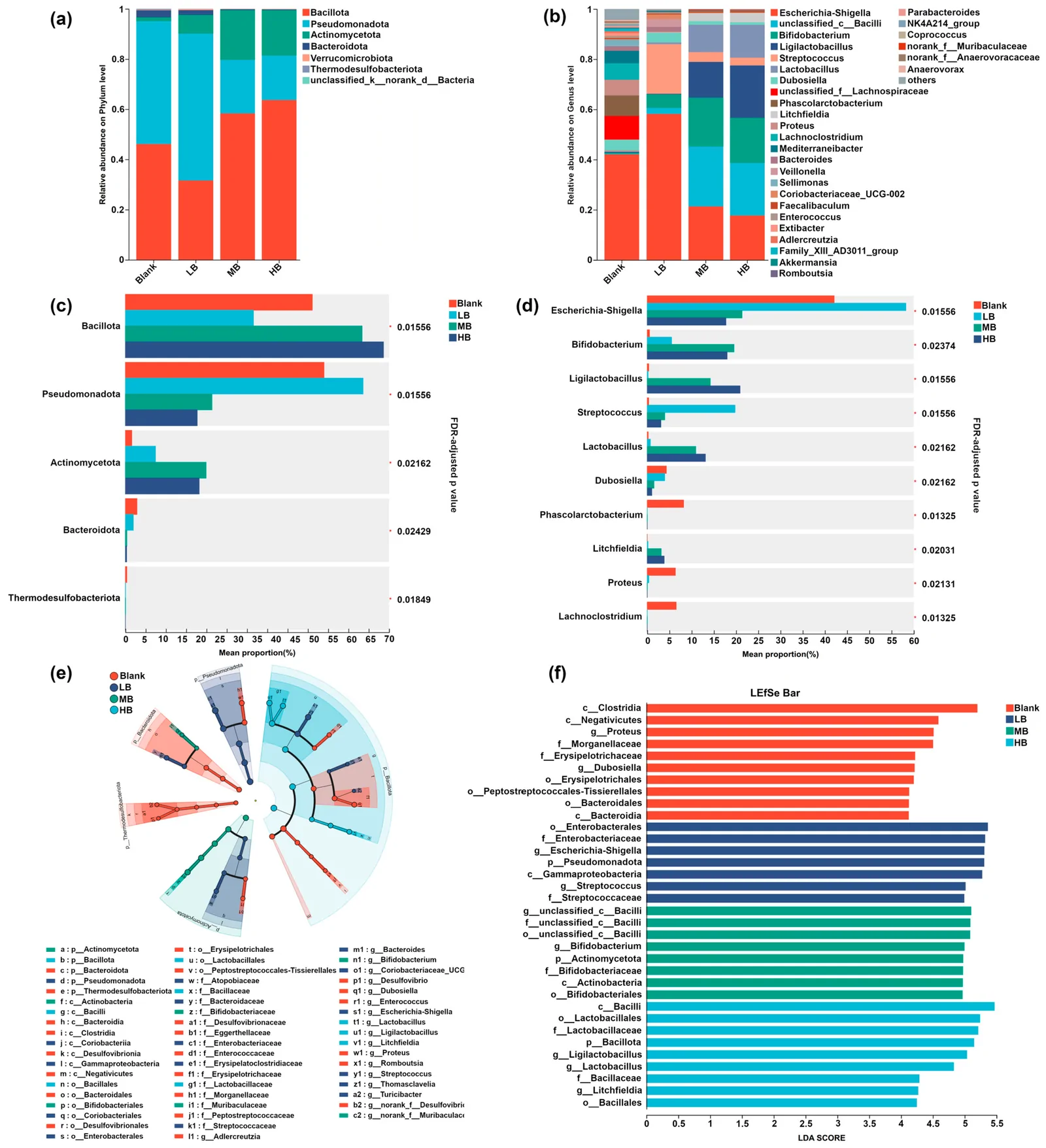
Effects of different BSP doses on microbial community composition during in vitro fermentation: (**a**) relative abundance of dominant bacterial phyla; (**b**) relative abundance of dominant bacterial genera; (**c**) Kruskal–Wallis analysis of differential bacterial phyla; (**d**) Kruskal–Wallis analysis of differential bacterial genera; (**e**) LEfSe cladogram showing group-specific microbial biomarkers; (**f**) LDA score distribution of discriminative taxa identified by LEfSe (LDA score > 4.0). Analyses were based on samples from five separate fermentation tubes per group, all inoculated from the same pooled fecal sample.

At the phylum level (Figure 4a), Bacillota, Pseudomonadota, and Actinomycetota were the dominant phyla across all groups. BSP supplementation markedly altered their relative abundances. With increasing BSP dose, the relative abundance of Bacillota increased in the MB and HB groups, whereas Pseudomonadota showed a decreasing trend. Actinomycetota was also more abundant in the MB and HB groups. Kruskal–Wallis analysis (Figure 4c) identified significant differences among groups in Bacillota, Pseudomonadota, Actinomycetota, Bacteroidota, and Thermodesulfobacteriota (*p* < 0.05).

At the genus level (Figure 4b), the predominant genera included *Escherichia–Shigella*, *Bifidobacterium*, *Ligilactobacillus*, *Streptococcus*, and *Lactobacillus*. In LB, the relative abundances of *Escherichia–Shigella* and *Streptococcus* were 58.20% and 19.75%, respectively, compared with 42.07% and 0.32% in Blank. In MB and HB, *Bifidobacterium* reached 19.50% and 17.97%, respectively, compared with 0.51% in Blank. *Lactobacillus* increased from 0.20% in Blank to 10.91% in MB and 13.08% in HB, and *Ligilactobacillus* increased from 0.33% to 14.16% and 20.88%, respectively. Meanwhile, *Escherichia–Shigella* was lower in MB (21.31%) and HB (17.70%), while *Proteus* decreased from 6.32% in Blank to below 0.01% in both groups. Kruskal–Wallis analysis (Figure 4d) further identified *Escherichia–Shigella*, *Bifidobacterium*, *Ligilactobacillus*, *Streptococcus*, *Lactobacillus*, *Dubosiella*, *Phascolarctobacterium*, *Litchfieldia*, *Proteus*, and *Lachnoclostridium* as genera that differed significantly among the treatment groups.

LEfSe analysis identified discriminative taxa with LDA scores > 4.0 (Figure 4e,f). Distinct taxonomic signatures were observed among the four groups. The Blank group was mainly enriched in Clostridia, Negativicutes, *Proteus*, Morganellaceae, Erysipelotrichaceae, *Dubosiella*, Bacteroidales, and Bacteroidia. The LB group was characterized by enrichment of Pseudomonadota, Gammaproteobacteria, Enterobacterales, Enterobacteriaceae, *Escherichia–Shigella*, *Streptococcus*, and Streptococcaceae. In contrast, the MB group was enriched in Actinomycetota, Actinobacteria, Bifidobacteriales, Bifidobacteriaceae, and *Bifidobacterium*, with *Bifidobacterium* representing a characteristic genus of this group. The HB group was mainly enriched in Bacillota, Bacilli, Lactobacillales, Lactobacillaceae, *Lactobacillus*, *Ligilactobacillus*, Bacillales, Bacillaceae, and *Litchfieldia*.

Overall, these findings indicate that BSP promoted the proliferation of potentially beneficial bacteria while suppressing the growth of potentially harmful taxa, with more pronounced effects observed in the MB and HB groups.

### 3.6. Effects of Different BSP Doses on Metabolic Profiles During In Vitro Fermentation

**Figure 5:**
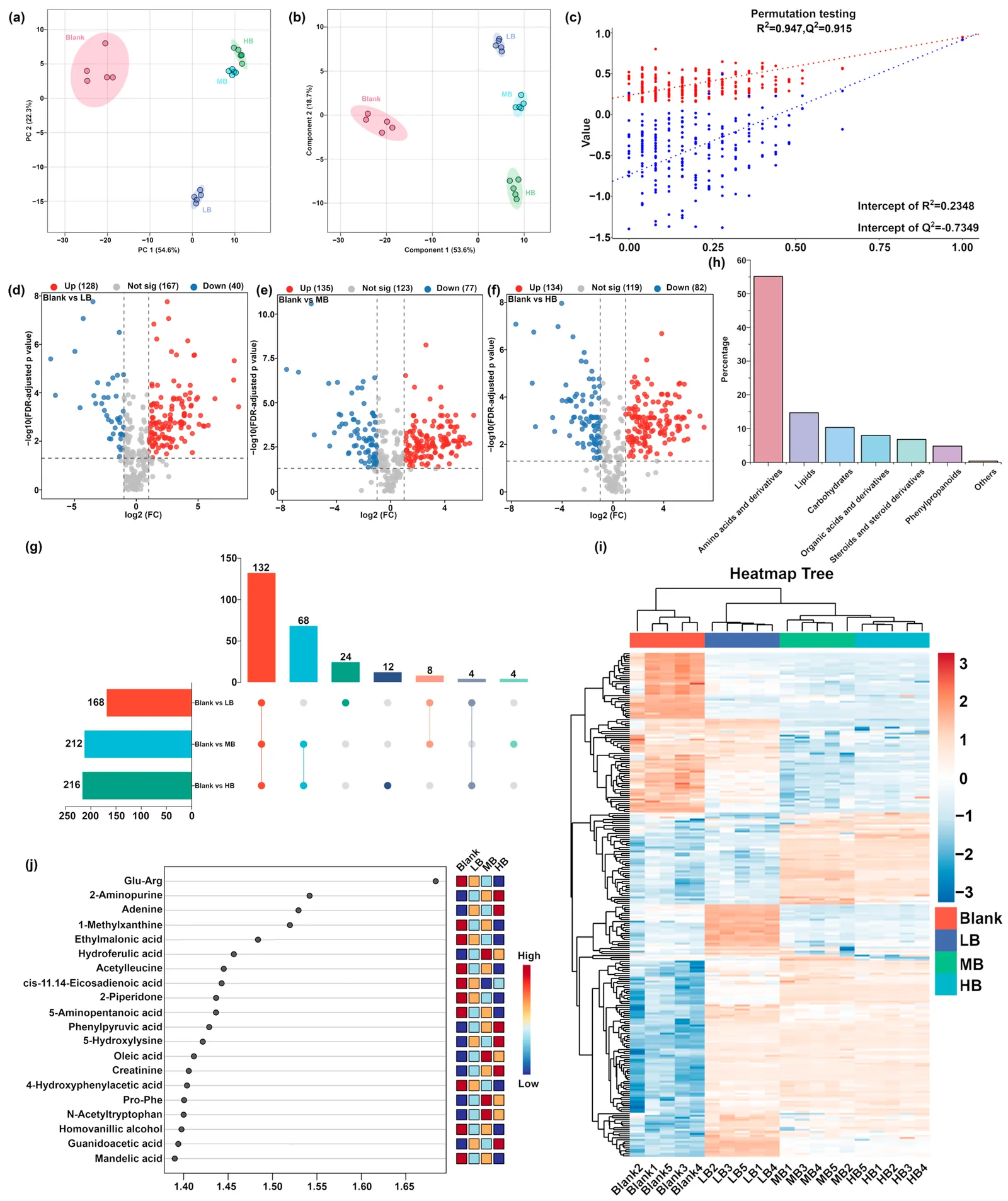
Effects of different BSP doses on metabolic profiles during in vitro fermentation: (**a**) principal component analysis (PCA) score plot; (**b**) partial least squares discriminant analysis (PLS-DA) score plot; (**c**) permutation test of the PLS-DA model; (**d**–**f**) volcano plots of differential metabolites for Blank vs. LB, Blank vs. MB, and Blank vs. HB, respectively; (**g**) UpSet plot showing shared and unique differential metabolites among the three comparisons; (**h**) chemical classification of differential metabolites; (**i**) hierarchical clustering heatmap of differential metabolites; (**j**) representative metabolites with VIP > 1 ranked according to their variable importance in projection (VIP) scores. Analyses were based on samples from five separate fermentation tubes per group, all inoculated from the same pooled fecal sample.

To evaluate the effects of different BSP doses on the overall metabolic profiles, PCA was first performed. As shown in Figure 5a, PC1 and PC2 explained 54.6% and 22.3% of the total variance, respectively, accounting for 76.9% cumulatively. The Blank, LB, MB, and HB groups were clearly separated in the two-dimensional score plot, while samples within each group clustered closely. PLS-DA further confirmed the separation of metabolic profiles among groups (Figure 5b), with the first two components explaining 53.6% and 18.7% of the variance, respectively. Model validation by permutation testing showed *R*^2^ and *Q*^2^ values of 0.947 and 0.915 for the original model, respectively (Figure 5c). The intercepts of the *R*^2^ and *Q*^2^ regression lines were 0.2348 and −0.7349, respectively, with the negative *Q*^2^ intercept indicating no apparent overfitting.

Using the Blank group as the reference, differential metabolites were screened in the LB, MB, and HB groups. With |log_2_FC| > 1 and FDR-adjusted *p* < 0.05 as the screening criteria, 168 differential metabolites were identified between the Blank and LB groups, including 128 increased and 40 decreased metabolites in LB relative to Blank (Figure 5d). A total of 212 differential metabolites were detected between Blank and MB, including 135 increased and 77 decreased metabolites (Figure 5e), whereas 216 were identified between Blank and HB, including 134 increased and 82 decreased metabolites (Figure 5f). UpSet analysis (Figure 5g) showed that 132 differential metabolites were shared among the Blank vs. LB, Blank vs. MB, and Blank vs. HB comparisons. In addition, 68 differential metabolites were shared by the MB- and HB-related comparisons. The Blank vs. LB, Blank vs. MB, and Blank vs. HB comparisons contained 24, 4, and 12 unique differential metabolites, respectively. These results indicate that BSP induced both common and dose-specific metabolic responses.

The differential metabolites were further classified into seven chemical categories (Figure 5h). Amino acids and derivatives represented the largest proportion, accounting for more than half of all differential metabolites, followed by lipids and carbohydrates. Other categories included organic acids and derivatives, steroids and steroid derivatives, and phenylpropanoids. Thus, the metabolic differences were dominated by amino acids and their derivatives, while also involving lipids, carbohydrates, and organic acids.

Hierarchical clustering analysis (Figure 5i) showed close clustering of samples within each group and distinct metabolite abundance patterns among the Blank, LB, MB, and HB groups. Several differential metabolites exhibited opposite abundance patterns between the Blank and BSP-treated groups, while distinct clustering patterns were also observed among the LB, MB, and HB groups. These results further demonstrate that different BSP doses generated distinct metabolic profiles.

Metabolites contributing substantially to group discrimination were further screened according to their variable importance in projection (VIP) values from the PLS-DA model (Table S1). As shown in Figure 5j, among the metabolites with VIP > 1, Glu-Arg had the highest VIP value, followed by 2-aminopurine, adenine, 1-methylxanthine, ethylmalonic acid, hydroferulic acid, and others, indicating their strong contributions to the discrimination among treatment groups.

Distinct group-specific abundance patterns were observed for these representative metabolites. Glu-Arg, 1-methylxanthine, ethylmalonic acid, acetylleucine, cis-11,14-eicosadienoic acid, 2-piperidone, 5-aminopentanoic acid, 4-hydroxyphenylacetic acid, homovanillic alcohol, and mandelic acid were relatively abundant in the Blank group. Hydroferulic acid, oleic acid, Pro-Phe, and N-acetyltryptophan were relatively abundant in the MB group, whereas 2-aminopurine, adenine, phenylpyruvic acid, 5-hydroxylysine, creatinine, and guanidoacetic acid were mainly enriched in the HB group. Overall, VIP-based screening further demonstrated dose-specific metabolite patterns and identified representative metabolites that contributed substantially to the metabolic differences among groups.

### 3.7. Effects of Different BSP Doses on KEGG Pathway Enrichment During In Vitro Fermentation

**Figure 6:**
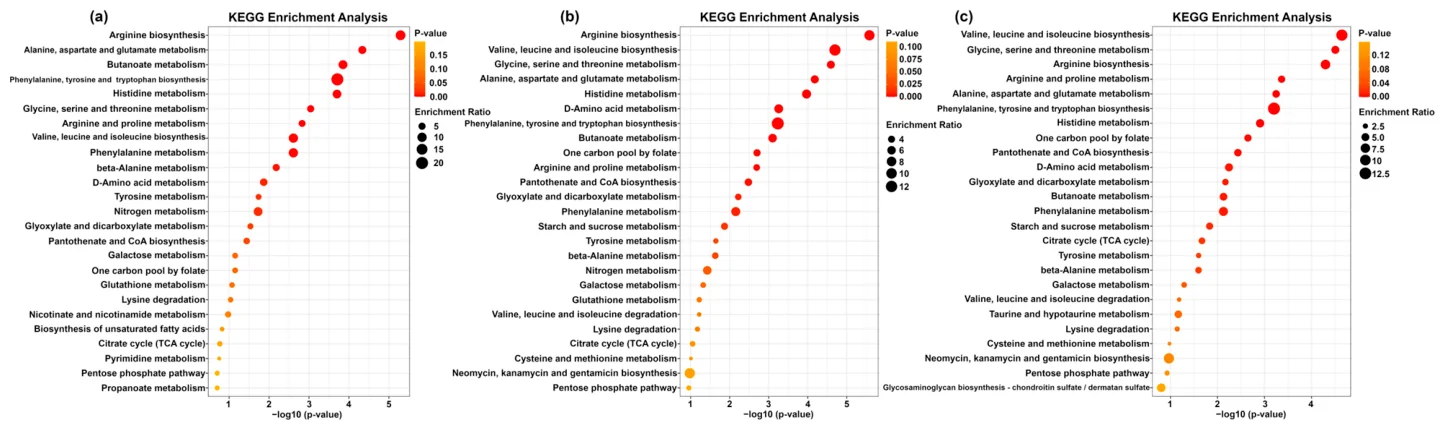
Effects of different BSP doses on KEGG pathway enrichment during in vitro fermentation: (**a**) Blank vs. LB; (**b**) Blank vs. MB; (**c**) Blank vs. HB. Bubble size represents the enrichment ratio, and bubble color indicates the corresponding *p* value.

KEGG pathway enrichment analysis was further performed on the differential metabolites identified in the different BSP groups (Figure 6a–c). Amino acid metabolism-related pathways were among the most prominently enriched pathways in all three comparisons (Blank vs. LB, Blank vs. MB, and Blank vs. HB), mainly including arginine biosynthesis, glycine, serine and threonine metabolism, alanine, aspartate and glutamate metabolism, and pathways related to phenylalanine, tyrosine, and tryptophan metabolism. Differences were also observed among the BSP dose groups in pathways associated with branched-chain amino acid metabolism, carbohydrate metabolism, and central carbon metabolism. In particular, the MB and HB groups showed more pronounced enrichment of starch and sucrose metabolism, galactose metabolism, and the tricarboxylic acid (TCA) cycle. Overall, BSP induced a set of common metabolic responses while also producing distinct dose-specific pathway enrichment patterns.

Collectively, these results indicate that microbial fermentation of BSP generated distinct dose-specific microbiota–metabolite responses, providing metabolic evidence for its modulation of the gut microbial ecosystem and potential prebiotic properties.

### 3.8. Associations Between Differential Genera and Metabolites During In Vitro Fermentation

**Figure 7:**
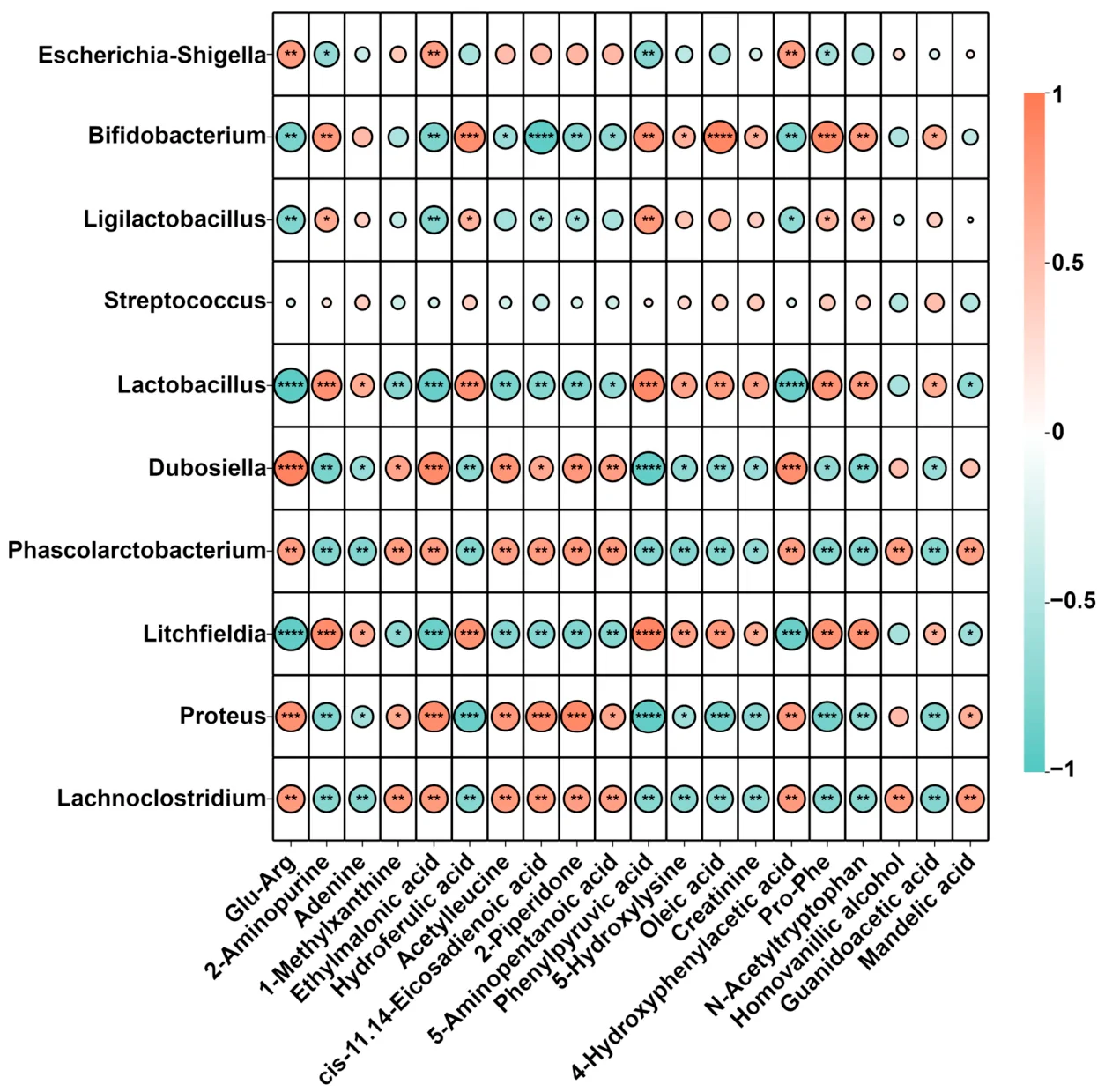
Correlations between differential bacterial genera and representative metabolites during in vitro fermentation with different BSP doses. Spearman correlation analysis was performed between the major differential genera and representative differential metabolites. Circle color represents the direction of correlation, with red indicating positive correlations and blue-green indicating negative correlations, while circle size reflects the absolute value of the correlation coefficient. Asterisks indicate statistical significance (* *p* < 0.05, ** *p* < 0.01, *** *p* < 0.001, and **** *p* < 0.0001).

To further investigate the relationships between microbial and metabolic changes, Spearman correlation analysis was performed between the major differential genera and representative differential metabolites. As shown in Figure 7, *Proteus*, *Dubosiella*, *Phascolarctobacterium*, and *Lachnoclostridium* were mainly positively correlated with Glu-Arg, 5-aminopentanoic acid, 2-piperidone, and 4-hydroxyphenylacetic acid. In contrast, *Bifidobacterium*, *Lactobacillus*, *Ligilactobacillus*, and *Litchfieldia* were mainly positively correlated with hydroferulic acid, phenylpyruvic acid, oleic acid, Pro-Phe, and N-acetyltryptophan, while showing significant negative correlations with Glu-Arg, 5-aminopentanoic acid, and 4-hydroxyphenylacetic acid. In addition, *Escherichia–Shigella* was positively correlated with several metabolites enriched in the Blank group, whereas *Streptococcus* showed relatively weak correlations with most metabolites.

Overall, clear associations were observed between the major differential genera and representative metabolites, indicating that BSP-induced changes in microbial community composition were closely associated with alterations in the metabolic profile.

## 4. Discussion

The present study evaluated the antioxidant properties of BSP during simulated gastrointestinal digestion and investigated how different BSP doses modulated gut microbial composition and metabolic profiles during in vitro fermentation. Overall, BSP retained appreciable antioxidant potential throughout digestion and elicited distinct, dose-specific shifts in both microbial community structure and metabolic characteristics. These findings highlight the importance of dose selection in evaluating BSP as a potential functional food ingredient.

Gastrointestinal digestion can modify the physicochemical properties and biological activities of polysaccharides, potentially influencing their subsequent microbial utilization in the colon. Total and reducing sugar content of BSP remained relatively stable during oral digestion, whereas gastric digestion decreased total sugar content and increased reducing sugar content. Similar increases in reducing sugar content, accompanied by lower molecular weights, were reported for *Dendrobium officinale* and *Polygonatum cyrtonema* polysaccharides after gastric digestion [7,28]. These changes may reflect partial cleavage of glycosidic linkages under acidic conditions, exposing additional reducing ends. During intestinal digestion, total sugar content decreased further, but reducing sugar content declined in contrast to the increases reported for these polysaccharides. BSP also showed distinct antioxidant responses: ABTS^+^ and DPPH scavenging activities decreased during gastric digestion and recovered during intestinal digestion, whereas ·OH scavenging activity increased throughout. Divergent responses have also been reported for Chinese yam polysaccharides, with increased DPPH scavenging activity but decreased reducing power during intestinal digestion [29]. These differences may reflect changes in polysaccharide conformation and the accessibility of antioxidant groups, together with the distinct reaction mechanisms of the antioxidant assays. Overall, BSP retained appreciable antioxidant potential during digestion, although further structural characterization is needed to clarify the molecular basis of these responses.

During fermentation, all BSP groups showed higher OD_600_ than the Blank group at 12 h, consistent with enhanced microbial growth. At 24–48 h, MB and HB maintained higher OD_600_ and lower pH than LB. Increased OD_600_ and acidification have also been reported during BSP fermentation, accompanied by rapid carbohydrate consumption [11]. The more sustained fermentation responses at medium and high doses may reflect greater initial availability of fermentable substrates. Dose-related changes have also been observed during type 2 resistant starch fermentation, with higher doses increasing short-chain fatty acid production and altering microbial community composition [14]. The limited changes in OD_600_ and pH from 24 to 48 h may reflect reduced availability of readily fermentable substrates and growth constraints under acidic conditions. Similar late-stage stabilization was reported during *Volvaria volvacea* polysaccharide fermentation [30]. These findings suggest that differences in substrate supply and acidification may contribute to the dose-specific microbial community shifts observed here.

BSP reduced microbial richness at all three doses, whereas diversity and community composition varied with dose. MB and HB had higher Shannon indices than LB, and their community compositions were more similar to each other than to those of LB or Blank. Similarly, *Poria cocos* polysaccharides altered Shannon and Simpson indices and community structure during fermentation, although richness did not differ significantly [31]. These observations highlight the importance of interpreting richness alongside community composition. Notably, microorganisms differ markedly in their capacity to utilize polysaccharides. Qu et al. attributed differences in the utilization of 20 medicinal polysaccharides by *Bacteroides* and *Parabacteroides* species to variation in carbohydrate-active enzyme profiles [32]. Such substrate selectivity may explain differences in the enrichment of particular taxa across BSP doses.

At the genus level, LB was characterized by high relative abundances of *Escherichia–Shigella* and *Streptococcus*, whereas MB and HB had lower relative abundances of *Escherichia–Shigella* and *Proteus* than Blank. These differences may have functional implications, as certain *Escherichia coli* strains have been shown to aggravate intestinal inflammation in mice [33], while *Proteus mirabilis* can induce epithelial inflammatory responses and exacerbate colonic inflammation [34]. *Streptococcus* enrichment may also reflect its involvement in carbohydrate fermentation, consistent with observations from previous BSP fermentation work [11]. MB was characterized by *Bifidobacterium* enrichment, with potential benefits for intestinal protection. For example, the indole-3-lactic acid-producing strain *Bifidobacterium bifidum* ZL.1 increased fecal indole-3-lactic acid levels, enhanced colonic MUC2 expression, and reduced inflammatory cytokines in mice with chronic kidney disease [35]. HB was characterized by *Lactobacillus* and *Ligilactobacillus*, which include strains with documented effects on epithelial integrity and inflammatory regulation. *Lactobacillus gasseri* ATCC 33323 enhanced E-cadherin expression and alleviated dextran sulfate sodium (DSS)-induced colitis [36]. *Ligilactobacillus murinus* also reduced intestinal barrier injury, with macrophage-derived IL-10 implicated in its protective effects [37]. Overall, medium and high BSP doses favored potentially beneficial taxa, suggesting that BSP may support intestinal health through microbial metabolite production.

The distinct microbial communities across BSP doses may reflect differences in substrate availability. Different initial BSP concentrations may affect both the amount and persistence of accessible carbon resources, influencing competition among taxa with different substrate requirements. In mice, inulin dosage affected the rate of microbial succession and the composition of responding taxa [12]. Cross-feeding can also support selective bacterial enrichment by supplying metabolites to bacteria unable to utilize the original substrate [38]. In the present study, the lower late-stage OD_600_ in LB contrasted with the sustained microbial growth and stronger acidification observed in MB and HB. Differences in substrate supply and acidification could therefore influence microbial competition and cross-feeding during BSP fermentation. Such interactions may explain why MB was characterized by *Bifidobacterium*, whereas HB favored *Lactobacillus* and *Ligilactobacillus*, rather than all responsive taxa increasing proportionally with BSP dose.

Metabolomic analysis revealed both shared and dose-specific responses to BSP fermentation. Amino acids and their derivatives accounted for more than half of the differential metabolites, with amino acid-related pathways enriched across all three doses. Similar changes in amino acid metabolism have been reported during in vitro fermentation of *Flammulina velutipes* polysaccharides [39]. Starch and sucrose metabolism were enriched in MB and HB, and the TCA cycle was additionally enriched in HB. Enrichment of starch and sucrose metabolism has likewise been observed during *Volvaria volvacea* polysaccharide fermentation [30]. Specific metabolites can serve as biochemical indicators for monitoring biological responses to interventions [40]. Examining the dose-associated metabolites may therefore help characterize BSP fermentation and its potential functional relevance.

Among the annotated metabolites, hydroferulic acid and oleic acid were relatively enriched in MB, whereas phenylpyruvic acid was more abundant in HB. Hydroferulic acid, also known as dihydroferulic acid, can arise from microbial reduction of ferulic acid [41]. It has been shown to reduce oxidative stress and apoptosis in hydrogen peroxide-exposed intestinal epithelial cells [42]. Oleic acid provides an additional link to immune regulation. Pompura et al. found that it enhanced human regulatory T-cell suppressive function by promoting fatty acid oxidation and increasing FOXP3 expression [43]. These findings suggest potential epithelial and immunomodulatory relevance for the MB-associated metabolites. In HB, phenylpyruvic acid points to altered phenylalanine metabolism. This intermediate can be converted to phenyllactic acid, which has antimicrobial activity [44], although precursor accumulation alone does not establish increased downstream production. These metabolite patterns provide a functional context for interpreting the distinct microbial responses to BSP dose.

Correlation analysis provided additional insight into the coupling between microbial restructuring and metabolic changes. *Bifidobacterium*, *Lactobacillus*, and *Ligilactobacillus* correlated positively with hydroferulic acid and phenylpyruvic acid; *Bifidobacterium* and *Lactobacillus* also correlated positively with oleic acid. The association with phenylpyruvic acid may reflect the capacity of some *Bifidobacterium* species to metabolize aromatic amino acids through aromatic pyruvic acid intermediates [45]. Conversely, *Proteus* and several other genera showed negative correlations with these metabolites and positive correlations with Glu-Arg, 5-aminopentanoic acid, and 4-hydroxyphenylacetic acid. These opposing patterns suggest differences in amino acid metabolism among the microbial communities. Carbohydrate availability can redirect microbial tryptophan metabolism through interspecies interactions [46], suggesting that substrate supply may contribute to these differences. Together, these correlations support coordinated microbial and metabolic responses to BSP dose, although they do not identify the taxa responsible for metabolite production.

Overall, BSP retained antioxidant potential during simulated digestion and elicited distinct microbial and metabolic responses across doses during in vitro fermentation. However, the dose-specific responses were characterized using a single pooled rat fecal inoculum, which precluded assessment of inter-individual variability and reproducibility across donors. Effects on intestinal tissue function were also not directly evaluated. A BSP-free digestion blank was not included, so contributions from digestive enzymes and bile salts to the sugar and antioxidant assays could not be distinguished. Future studies incorporating matched digestion blanks and independent human fecal inocula should assess these background contributions and the reproducibility of the dose responses. Intestinal epithelial or tissue models and in vivo studies could evaluate the effects of BSP fermentation products on barrier integrity and inflammatory responses. Combining BSP structural characterization with metagenomics, targeted metabolomics, and cultivation of key strains could clarify the mechanisms underlying these microbial and metabolic responses. These findings provide a basis for further evaluation of BSP as a potential functional food ingredient.

## 5. Conclusions

This study investigated the digestive characteristics of BSP and the effects of different BSP doses on gut microbial and metabolic profiles during in vitro fermentation. BSP underwent changes in sugar content while retaining appreciable antioxidant activity during simulated gastrointestinal digestion. In vitro fermentation with a pooled rat fecal inoculum revealed dose-specific microbial and metabolic responses to BSP. MB was characterized by enrichment of *Bifidobacterium*, whereas HB favored *Lactobacillus* and *Ligilactobacillus*. These shifts were accompanied by differences in amino acid, carbohydrate, and central carbon metabolism. Microbiota–metabolite correlations linked the observed responses but did not establish causal relationships. These findings provide a basis for further evaluation of BSP as a potential functional food ingredient. Studies using independent human fecal inocula and host models are needed to assess reproducibility and physiological relevance.

## Figures and Tables

**Table 1:** Changes in the total sugar and reducing sugar content of BSP during in vitro simulated gastrointestinal digestion.

Digestion Phase	Digestion Time	Total Sugar Concentration (mg/mL)	Reducing Sugar Concentration (mg/mL)
SSF	0 min	0.674 ± 0.016 A *	0.498 ± 0.005 B
	1 min	0.669 ± 0.004 A	0.481 ± 0.014 B
	2 min	0.670 ± 0.003 A	0.483 ± 0.012 B
SGF	1 h	0.599 ± 0.009 B	0.533 ± 0.009 A
	2 h	0.591 ± 0.017 B	0.534 ± 0.014 A
SIF	1 h	0.533 ± 0.010 C	0.428 ± 0.002 C
	2 h	0.528 ± 0.004 C	0.428 ± 0.006 C

* Values are expressed as the mean ± standard deviation (SD). Different uppercase letters within the same column indicate significant differences among digestion time points (*p* < 0.05), as determined by one-way ANOVA followed by Tukey’s multiple-comparison test.

## Data Availability

The original contributions presented in this study are included in the article/Supplementary Material. Further inquiries can be directed to the corresponding author.

## Supplementary Materials

The following supporting information can be downloaded at https://www.mdpi.com/article/10.3390/foods15213803/s1, Table S1: Relative abundance of representative metabolites among different BSP dose groups during in vitro fermentation.
