# Supplementary material for: INFOGEST Digestion and In Vitro Rat Fecal Fermentation of Bletilla striata Polysaccharides: Digestive Stability and Dose-Specific Microbiota–Metabolite Responses
Source: Foods. 2026 Oct 23;15(21):3803. doi: 10.3390/foods15213803 (PMC13762231; doi:10.3390/foods15213803)
Supplement: Supplementary file 1 [file foods-15-03803-s001.zip › foods-4572424-supplementary.pdf]

INFOGEST Digestion and In Vitro Rat Fecal Fermentation of *Bletilla striata* Polysaccharides: Digestive Stability and Dose-Specific Microbiota–Metabolite Responses

Supplementary Material

Table S1. Relative abundance of representative metabolites among different BSP dose groups during in vitro fermentation

Table S1. Relative abundance of representative metabolites among different BSP dose groups during in vitro fermentation

Retention Time (s)	M/Z	Metabolite	Formula	CAS	Type	MS score	Level	Adduct	Mz_error	Rt_error	Group			
											Blank average	LB average	MB average	HB average
258.9	304.161	Glu-Arg	$C_{11}H_{21}N_5O_5$	7219-59-2	POS	3.93	Level 1	[M+H]+	1.9	3.6	$3.27\times10^{-4} \pm 3.72\times10^{-5}$	$1.57\times10^{-4} \pm 1.85\times10^{-5}$	$8.48\times10^{-5} \pm 9.35\times10^{-7}$	$7.69\times10^{-5} \pm 7.90\times10^{-6}$
73.3	134.047	2-Aminopurine	$C_5H_5N_5$	452-06-2	NEG	3.90	Level 1	[M-H]-	1.4	8.9	$2.67\times10^{-4} \pm 1.43\times10^{-4}$	$1.08\times10^{-3} \pm 1.92\times10^{-4}$	$1.28\times10^{-3} \pm 1.64\times10^{-4}$	$1.78\times10^{-3} \pm 1.01\times10^{-4}$
73.0	136.0614	Adenine	$C_5H_5N_5$	73-24-5	POS	3.92	Level 1	[M+H]+	2.8	3.5	$6.62\times10^{-4} \pm 1.89\times10^{-4}$	$6.41\times10^{-3} \pm 1.73\times10^{-4}$	$5.77\times10^{-3} \pm 5.18\times10^{-4}$	$9.56\times10^{-3} \pm 5.09\times10^{-4}$
41.9	165.0415	1-Methylxanthine	$C_6H_6N_4O_2$	6136-37-4	NEG	3.90	Level 1	[M-H]-	1.5	9	$9.15\times10^{-4} \pm 3.64\times10^{-4}$	$1.22\times10^{-4} \pm 1.80\times10^{-5}$	$1.58\times10^{-4} \pm 2.57\times10^{-5}$	$5.89\times10^{-5} \pm 1.03\times10^{-5}$
202.4	131.0348	Ethylmalonic acid	$C_5H_8O_4$	601-75-2	NEG	3.66	Level 1	[M-H]-	1.1	35.4	$7.42\times10^{-4} \pm 3.50\times10^{-4}$	$2.47\times10^{-4} \pm 2.18\times10^{-5}$	$2.10\times10^{-4} \pm 2.42\times10^{-5}$	$1.82\times10^{-4} \pm 1.95\times10^{-6}$
56.7	195.0659	Hydroferulic acid	$C_{10}H_{12}O_4$	1135-23-5	NEG	3.78	Level 1	[M-H]-	1.6	18.5	$1.95\times10^{-5} \pm 1.07\times10^{-5}$	$1.30\times10^{-4} \pm 1.37\times10^{-5}$	$2.51\times10^{-4} \pm 1.40\times10^{-5}$	$1.79\times10^{-4} \pm 5.01\times10^{-6}$
113.7	172.0978	Acetylleucine	$C_8H_{15}NO_3$	1188-21-2	NEG	3.88	Level 1	[M-H]-	0.8	10.6	$2.69\times10^{-3} \pm 9.81\times10^{-4}$	$6.60\times10^{-4} \pm 6.65\times10^{-5}$	$7.08\times10^{-4} \pm 5.41\times10^{-5}$	$5.52\times10^{-4} \pm 2.42\times10^{-5}$
18.5	307.2632	cis-11,14-Eicosadienoic acid	$C_{20}H_{36}O_2$	5598-38-9	NEG	3.93	Level 1	[M-H]-	3.3	2.3	$6.88\times10^{-4} \pm 1.09\times10^{-4}$	$3.68\times10^{-4} \pm 1.57\times10^{-5}$	$2.31\times10^{-4} \pm 2.95\times10^{-5}$	$3.11\times10^{-4} \pm 3.53\times10^{-5}$
26.0	100.0754	2-Piperidone	C_5H_9NO	27154-43-4	POS	3.90	Level 1	[M+H]+	3.6	5.1	$2.89\times10^{-1} \pm 3.06\times10^{-2}$	$4.05\times10^{-3} \pm 1.05\times10^{-3}$	$3.58\times10^{-3} \pm 2.13\times10^{-3}$	$2.29\times10^{-3} \pm 1.83\times10^{-4}$
228.8	118.086	5-Aminopentanoic acid	$C_5H_{11}NO_2$	660-88-8	POS	3.91	Level 1	[M+H]+	2.5	4.1	$5.62\times10^{-2} \pm 1.23\times10^{-2}$	$2.73\times10^{-3} \pm 3.79\times10^{-4}$	$2.57\times10^{-3} \pm 1.05\times10^{-4}$	$1.98\times10^{-3} \pm 5.98\times10^{-5}$
106.8	163.0399	Phenylpyruvic acid	$C_9H_8O_3$	156-06-9	NEG	3.87	Level 1	[M-H]-	1.1	13.3	$2.33\times10^{-5} \pm 1.64\times10^{-5}$	$3.56\times10^{-4} \pm 6.68\times10^{-6}$	$3.86\times10^{-4} \pm 2.17\times10^{-6}$	$4.51\times10^{-4} \pm 1.22\times10^{-5}$
300.4	163.1074	5-Hydroxylysine	$C_6H_{14}N_2O_3$	1190-94-9	POS	3.88	Level 1	[M+H]+	2.4	8.4	$2.83\times10^{-4} \pm 6.58\times10^{-5}$	$9.54\times10^{-4} \pm 2.76\times10^{-5}$	$8.89\times10^{-4} \pm 9.89\times10^{-5}$	$1.01\times10^{-3} \pm 4.43\times10^{-5}$
20.0	281.2479	Oleic acid	$C_{18}H_{34}O_2$	68412-07-7	NEG	3.91	Level 1	[M-H]-	2.4	6.3	$3.33\times10^{-2} \pm 6.97\times10^{-3}$	$6.62\times10^{-2} \pm 6.11\times10^{-3}$	$8.65\times10^{-2} \pm 3.08\times10^{-3}$	$7.67\times10^{-2} \pm 1.36\times10^{-3}$
89.8	112.0515	Creatinine	$C_4H_7N_3O$	60-27-5	NEG	3.94	Level 1	[M-H]-	1.1	4.8	$2.19\times10^{-5} \pm 2.26\times10^{-5}$	$2.13\times10^{-4} \pm 2.46\times10^{-4}$	$3.05\times10^{-4} \pm 1.38\times10^{-4}$	$3.10\times10^{-4} \pm 8.07\times10^{-5}$

Retention Time (s)	M/Z	Metabolite	Formula	CAS	Type	MS score	Level	Adduct	Mz_error	Rt_error	Group			
											Blank average	LB average	MB average	HB average
112.3	151.0399	4-Hydroxyphenylacetic acid	$C_8H_8O_3$	156-38-7	NEG	3.94	Level 1	[M-H]-	0.7	3.1	$3.49\times10^{-4} \pm 7.49\times10^{-5}$	$2.51\times10^{-4} \pm 1.03\times10^{-5}$	$2.38\times10^{-4} \pm 3.66\times10^{-6}$	$2.28\times10^{-4} \pm 4.26\times10^{-6}$
148.5	263.1383	Pro-Phe	$C_{14}H_{18}N_2O_3$	13589-02-1	POS	3.93	Level 1	[M+H]+	2.7	1.1	$6.33\times10^{-6} \pm 2.31\times10^{-6}$	$3.39\times10^{-4} \pm 1.77\times10^{-5}$	$1.62\times10^{-3} \pm 1.64\times10^{-4}$	$4.86\times10^{-4} \pm 5.66\times10^{-5}$
116.3	245.093	N-Acetyltryptophan	$C_{13}H_{14}N_2O_3$	1218-34-4	NEG	3.88	Level 1	[M-H]-	0.8	5.2	$4.92\times10^{-5} \pm 1.98\times10^{-5}$	$8.42\times10^{-5} \pm 1.34\times10^{-5}$	$9.80\times10^{-5} \pm 1.14\times10^{-5}$	$9.92\times10^{-5} \pm 2.88\times10^{-6}$
36.8	149.0605	Homovanillic alcohol	$C_9H_{12}O_3$	2380-78-1	NEG	3.80	Level 1	[M-H2O-H]-	1.3	20.6	$7.00\times10^{-3} \pm 1.46\times10^{-3}$	$1.40\times10^{-3} \pm 5.80\times10^{-5}$	$1.47\times10^{-3} \pm 8.02\times10^{-6}$	$1.34\times10^{-3} \pm 3.94\times10^{-5}$
211.7	118.0609	Guanidoacetic acid	$C_3H_7N_3O_2$	352-97-6	POS	3.95	Level 1	[M+H]+	2	0.6	$3.70\times10^{-5} \pm 1.32\times10^{-5}$	$2.96\times10^{-4} \pm 8.49\times10^{-5}$	$2.98\times10^{-4} \pm 3.10\times10^{-5}$	$2.98\times10^{-4} \pm 7.46\times10^{-6}$
22.0	151.0399	Mandelic acid	$C_8H_8O_3$	611-71-2	NEG	3.85	Level 1	[M-H]-	1.2	15.2	$9.78\times10^{-4} \pm 4.38\times10^{-4}$	$9.29\times10^{-5} \pm 2.14\times10^{-5}$	$1.01\times10^{-4} \pm 2.53\times10^{-5}$	$9.37\times10^{-5} \pm 1.72\times10^{-5}$

Values are presented as mean ± SD for five fermentation tubes per group, all inoculated from a single pooled fecal sample from 10 rats
